# Supplementary material for: OH spectator at IrMo intermetallic narrowing activity gap between alkaline and acidic hydrogen evolution reaction
Source: Nat Commun. 2022 Sep 20;13:5497. doi: 10.1038/s41467-022-33216-w (PMC9489878; doi:10.1038/s41467-022-33216-w)
Supplement: Supplementary file 1 — Supplementary Information [file 41467_2022_33216_MOESM1_ESM.pdf]

Supplementary Information

OH Spectator at IrMo Intermetallic Narrowing Activity Gap between Alkaline and Acidic Hydrogen Evolution Reaction

Jiaxi Zhang,[1,†] Longhai Zhang,[1,†] Jiamin Liu,[1] Chengzhi Zhong,[1] Yuanhua Tu,[1] Peng Li,[2] Li Du,[1] Shengli Chen,[2,] and Zhiming Cui[1,*]*

[1] Guangdong Provincial Key Laboratory of Fuel Cell Technology, School of Chemistry and Chemical Engineering, South China University of Technology, Guangzhou 510641, China

[2] Hubei Key Laboratory of Electrochemical Power Sources, College of Chemistry and Molecular Sciences, Wuhan University, Wuhan 430072, China.

† These authors contributed equally.

* Corresponding Authors. E-mail: zmcui@scut.edu.cn; slchen@whu.edu.cn

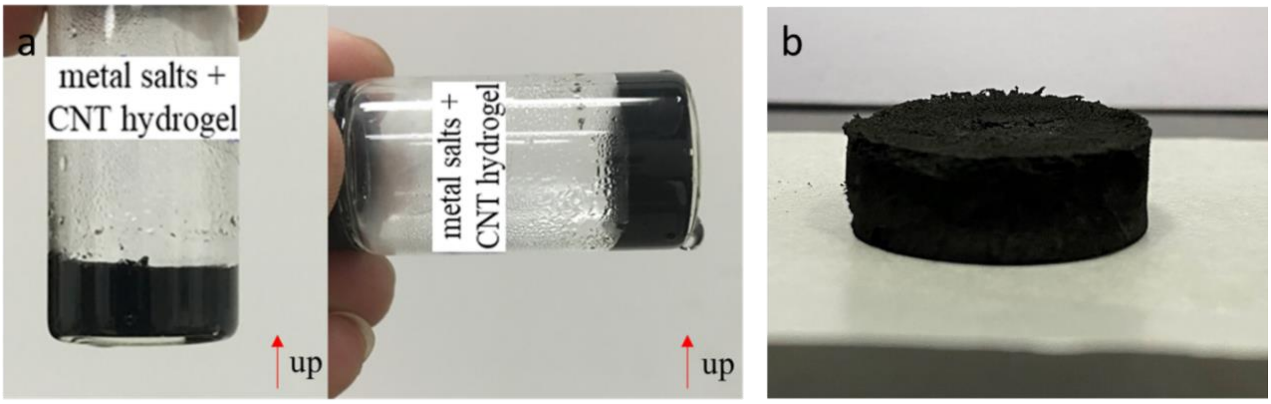

Supplementary Fig. 1 | Digital photographs of the precursors. a, The prepared metal salts/CNT hydrogel. **b**, Metal salts/CNT aerogel.

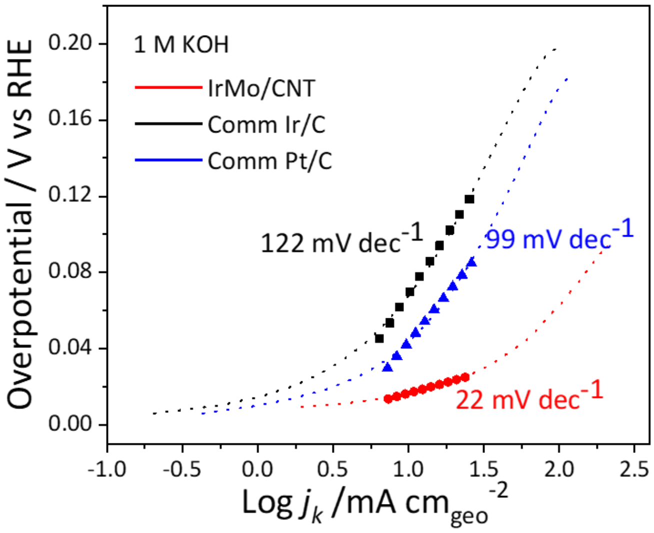

Supplementary Fig. 2 | HER Tafel slope analysis. Tafel plots in 1 M KOH for IrMo/CNT, commercial Ir/C and Pt/C.

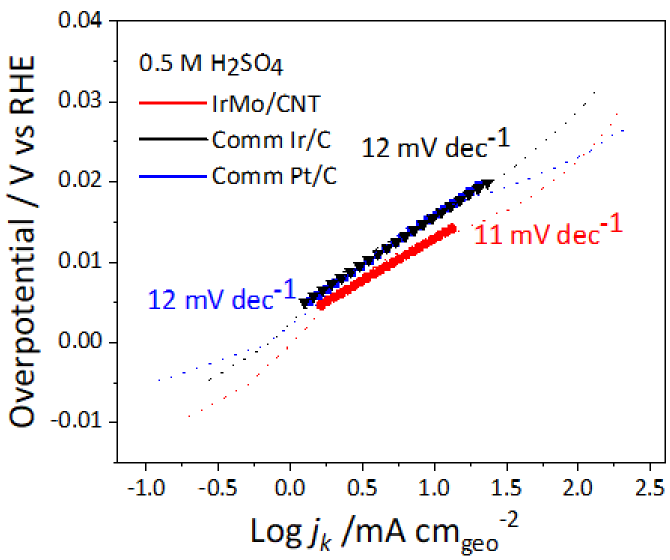

Supplementary Fig. 3 | HER Tafel slope analysis. Tafel plots for IrMo/CNT, commercial Ir/C and Pt/C in 0.5 M H_2SO_4.

Supplementary Note 1.

Due to the complex kinetics in alkaline HER, it is challenging to preserve the stable activity during long-term operation. The stability test of IrMo/CNT in 1 M KOH shows that the IrMo/CNT only exhibits a slight decrease of the activity after 10,000-cycle test; while the commercial Pt/C exhibits a heavy deterioration (supplementary Fig. 4-5). The cyclic voltammetry (CV) curves show that the hydrogen adsorption/desorption peaks coincide well before and after stability test for IrMo/CNT, implying a similar electrochemical surface area (ECSA) even after 10,000-cycle test (supplementary Fig. 6). This is consistent with the TEM images of IrMo/CNT before and after stability test (supplementary Fig. 7a, c), which clearly suggests no aggregation or dissolution of IrMo NPs during the long-term test. By contrast, the Pt/C shows a sharped decrease in the area of hydrogen adsorption/desorption peaks after 10,000-cycle test (supplementary Fig. 8), which should be ascribed to the dissolution and reaggregation of Pt NPs during the stability test and thereby leading to a heavy decrease of HER performance for Pt/C. This is consistent with the result of our previous work.[1] The stability tests of IrMo/CNT and Pt/C in acidic condition show the similar results with their stability test in alkaline condition. The IrMo/CNT exhibits highly stable performance and no dissolution and aggregation of IrMo NPs even after 10,000-cycle test in strong acidic

condition (supplementary Fig. 9, 10, 7b); while the Pt/C exhibits obvious decrease of HER activity and ECSA after the same period test (supplementary Fig. 11, 12).

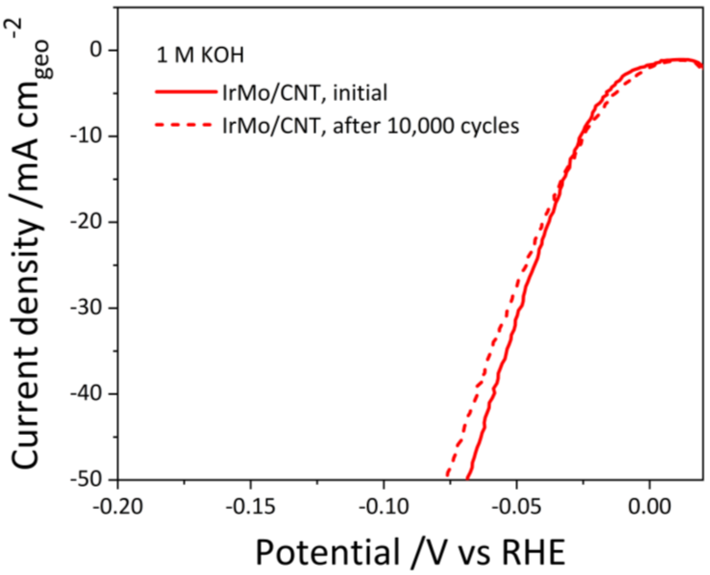

Supplementary Fig. 4 | HER stability test. HER LSV curves of IrMo/CNT before and after 10000 cycles in N_2-saturated 1 M KOH.

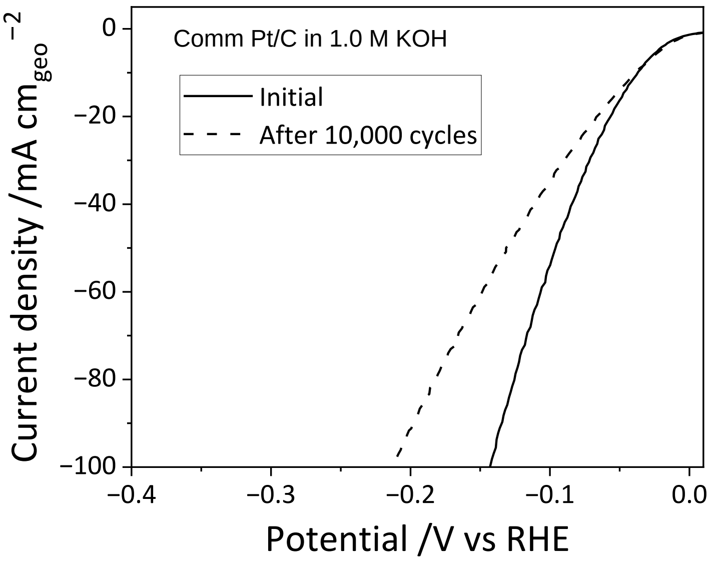

Supplementary Fig. 5 | HER stability test. HER LSV curves of Pt/C before and after 10000 cycles in N_2-saturated 1 M KOH.

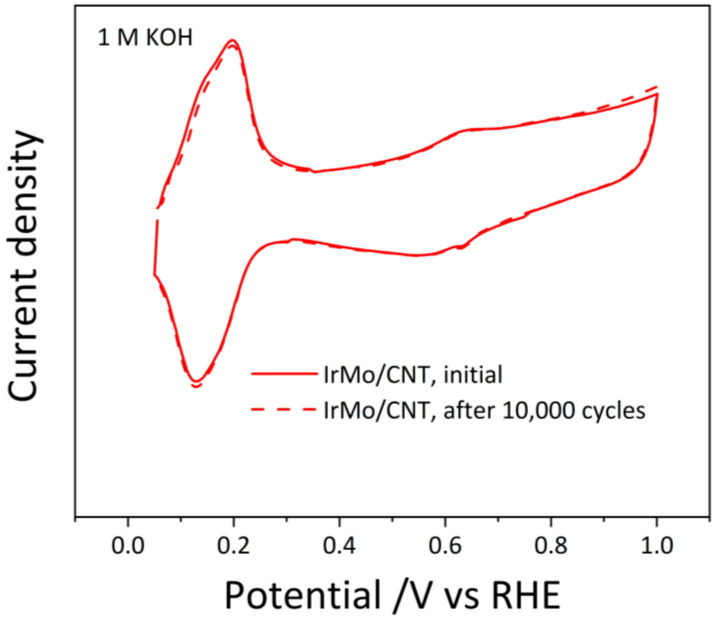

Supplementary Fig. 6 | H adsorption and desorption behavior. Cyclic voltammetry (CV) curves of IrMo/CNT before and after 10000-cycle HER stability test in N_2-saturated 1 M KOH.

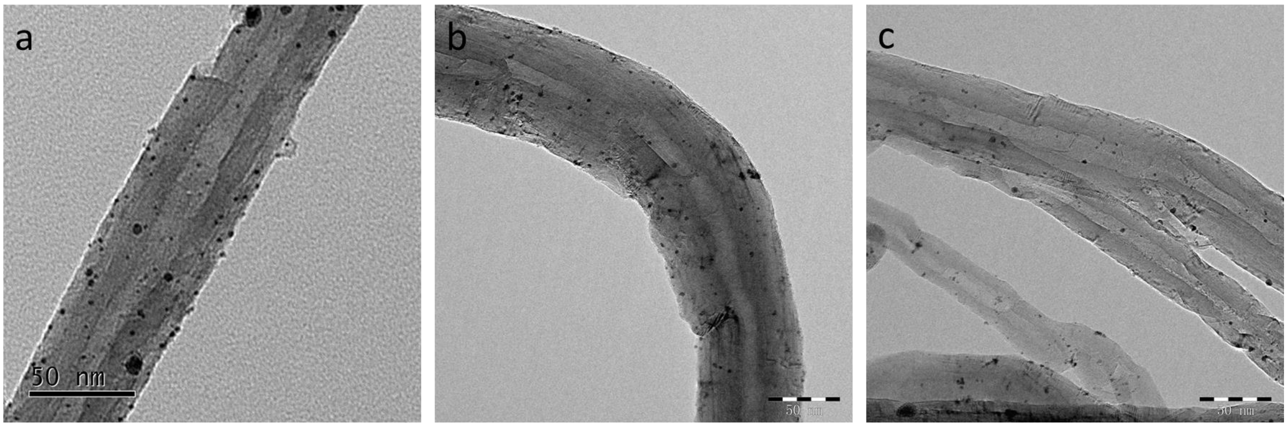

Supplementary Fig. 7 | TEM morphology. TEM images of IrMo/CNT: **a**, Pristine. After 10000-cycle HER stability test in **b**, 0.5 M H_2SO_4 and **c**, 1 M KOH.

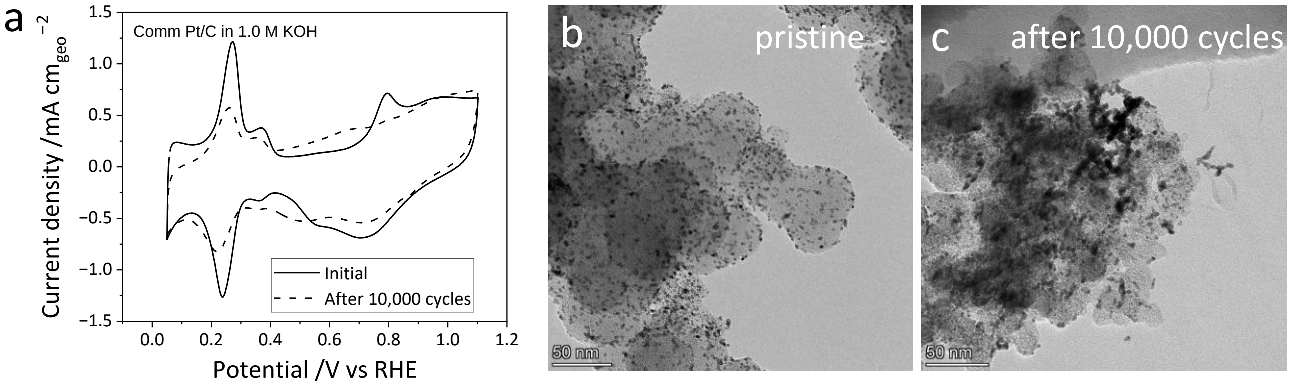

Supplementary Fig. 8 | Exposed surface and particle distribution of the Pt NPs in Pt/C. **a**, Cyclic voltammetry (CV) curves of Pt/C before and after 10000-cycle HER stability test in N_2-saturated 1 M KOH. TEM images of Pt/C: **b**, before and **c**, after 10,000 cycles measurement.

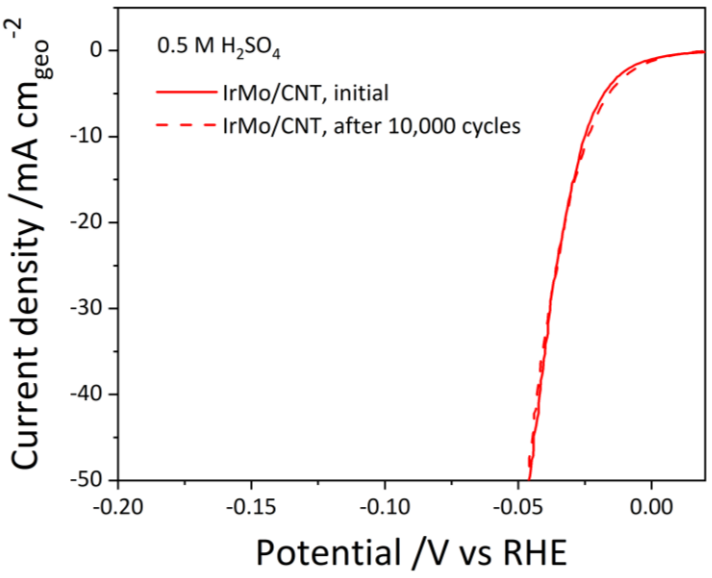

Supplementary Fig. 9 | HER stability test. HER LSV curves of IrMo/CNT before and after 10000 cycles in N_2-saturated 0.5 M H_2SO_4.

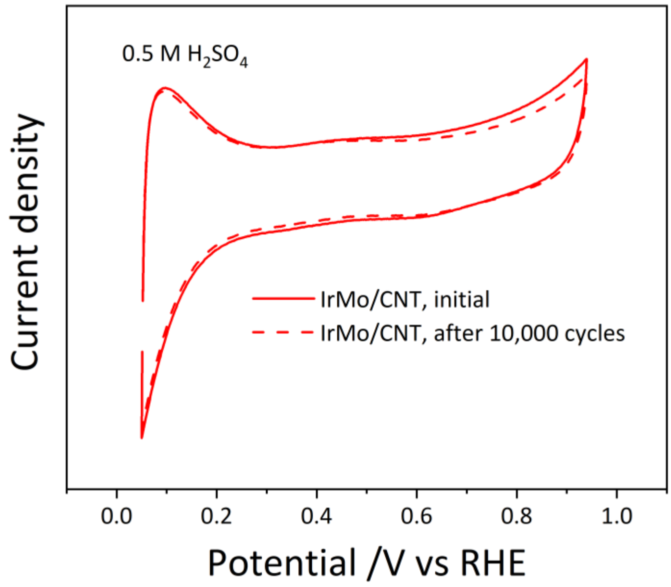

Supplementary Fig. 10 | H adsorption and desorption behavior. Cyclic voltammetry (CV) curves of IrMo/CNT before and after 10000-cycle HER stability test in N_2-saturated 0.5 M H_2SO_4.

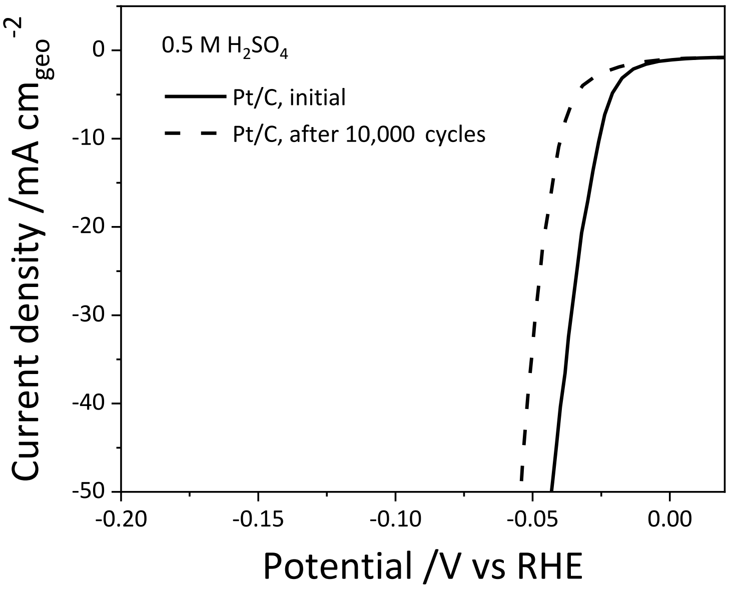

Supplementary Fig. 11 | HER stability test. HER LSV curves of Pt/C before and after 10000 cycles in N_2-saturated 0.5 M H_2SO_4.

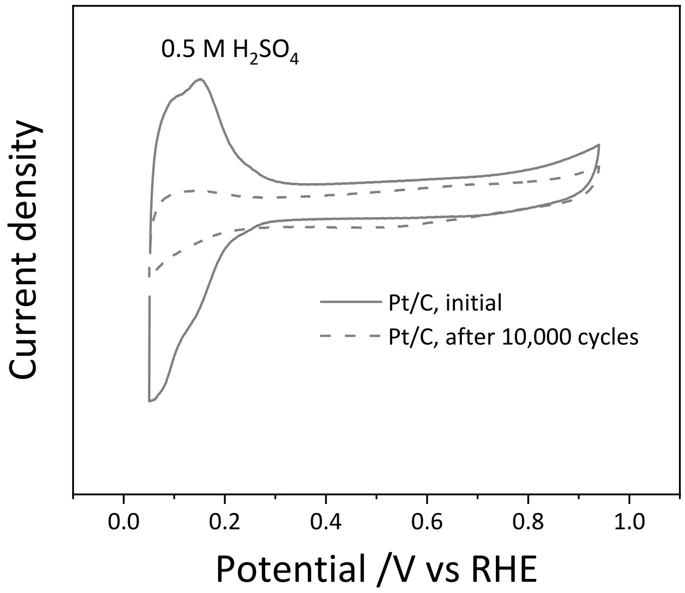

Supplementary Fig. 12 | H adsorption and desorption behavior. Cyclic voltammetry (CV) curves of Pt/C before and after 10000-cycle HER stability test in N_2-saturated 0.5 M H_2SO_4.

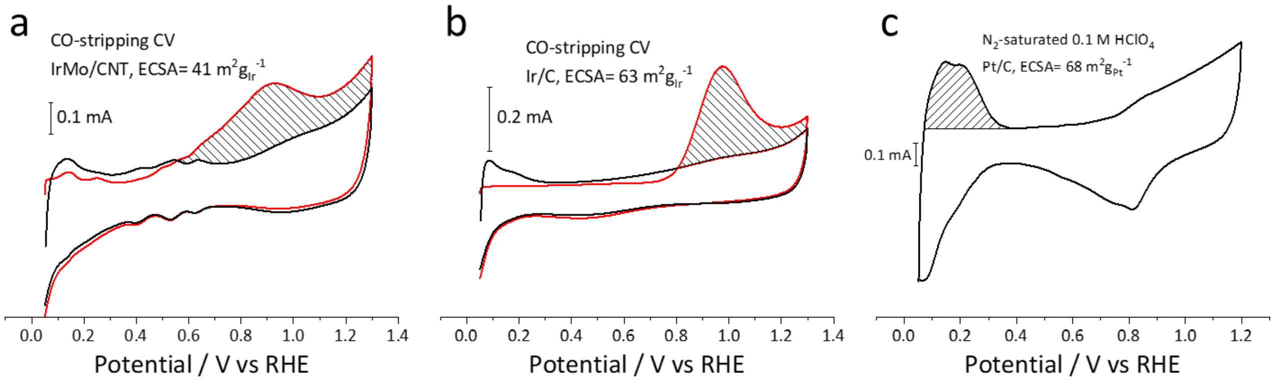

Supplementary Fig. 13 | ECSA measurement. CO-stripping CV curves of **a**, IrMo/CNT and **b**, Ir/C in 0.5 M H_2SO_4. **c**, CV curves of Pt/C in N_2-saturated 0.1 M $HClO_4$.

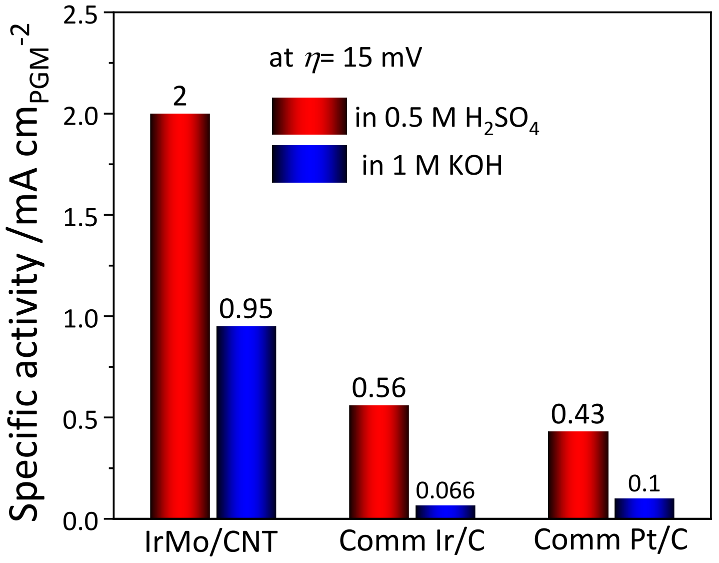

Supplementary Fig. 14 | Specific activity Comparison. The specific activity at overpotential of 15 mV of IrMo/CNT, Comm Ir/C and Pt/C in different media.

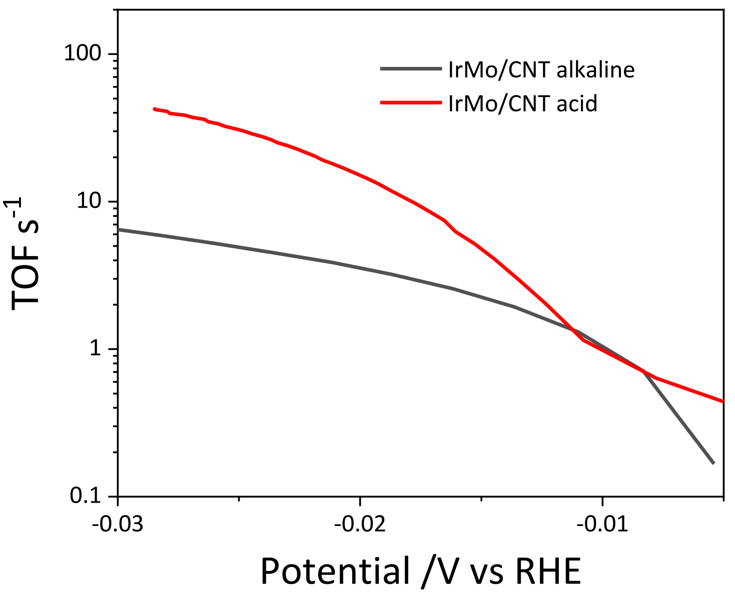

Supplementary Fig. 15 | TOF analysis. The TOF plots of IrMo/CNT as function with potential in acidic and alkaline electrolytes.

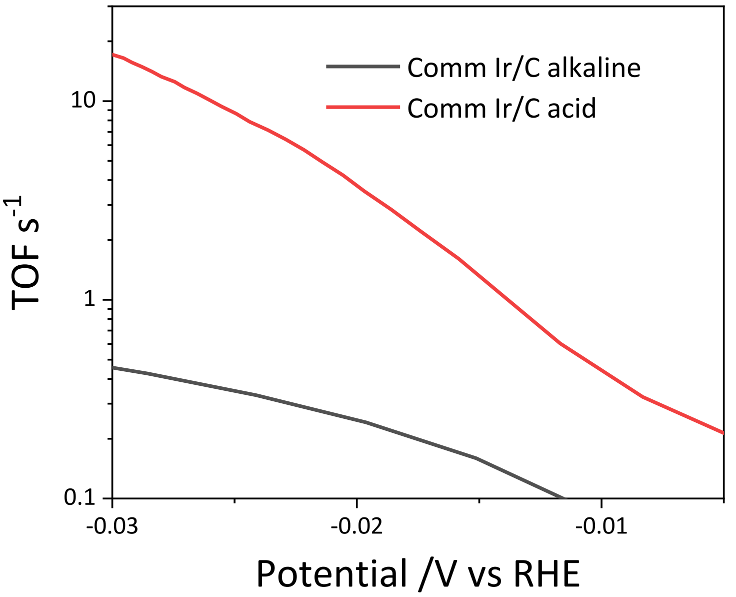

Supplementary Fig. 16 | TOF analysis. The TOF plots of commercial Ir/C as function with potential in acidic and alkaline electrolytes.

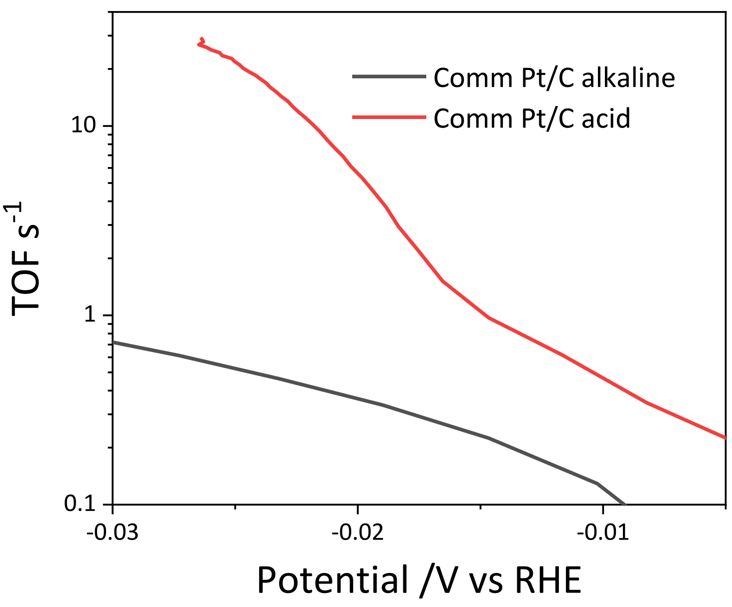

Supplementary Fig. 17 | TOF analysis. The TOF plots of commercial Pt/C as function with potential in acidic and alkaline electrolytes.

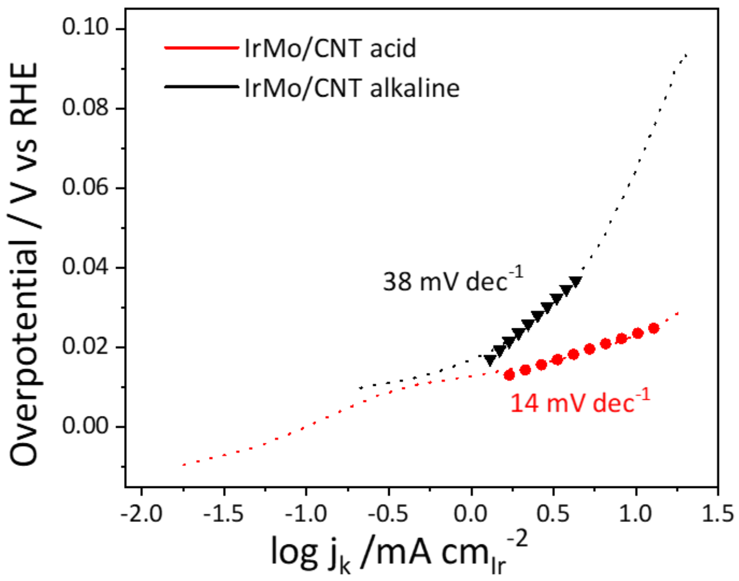

Supplementary Fig. 18 | Intrinsic Tafel plots. The Tafel plots with the kinetics current density normalized by ECSA of IrMo/CNT electrocatalysts in alkaline and acidic electrolytes.

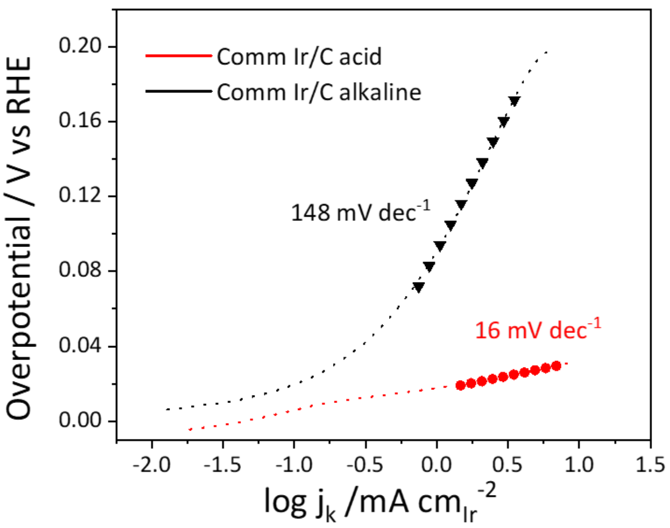

Supplementary Fig. 19 | Intrinsic Tafel plots. The Tafel plots with the kinetics current density normalized by ECSA of Ir/C electrocatalysts in alkaline and acidic electrolytes.

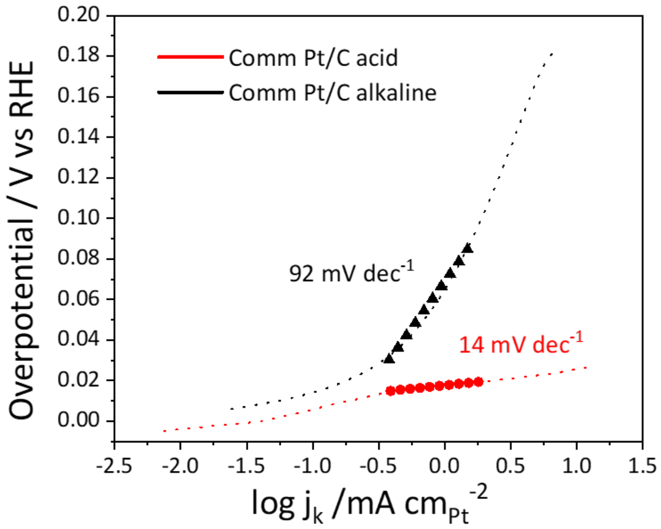

Supplementary Fig. 20 | Intrinsic Tafel plots. The Tafel plots with the kinetics current density normalized by ECSA of Pt/C electrocatalysts in alkaline and acidic electrolytes.

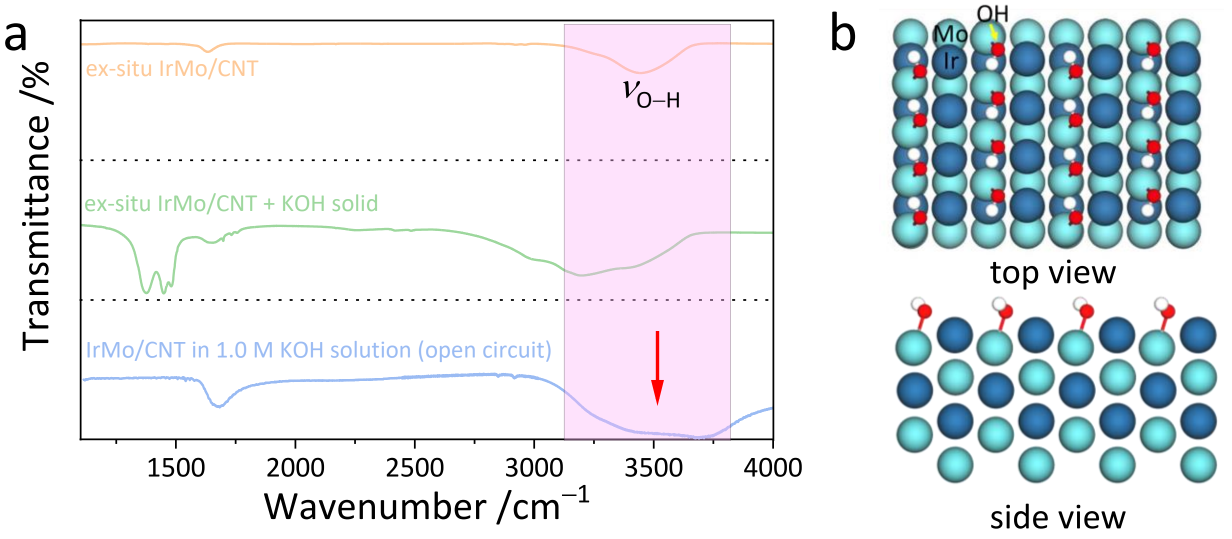

Supplementary Fig. 21 | In-situ FTIR and DFT model. a, FTIR spectra of ex-situ IrMo/CNT, IrMo/CNT in KOH solid and IrMo/CNT in 1.0 M KOH solution under open-circuit condition. **b**, IrMo (001) slab with a monolayer absorbed OH.

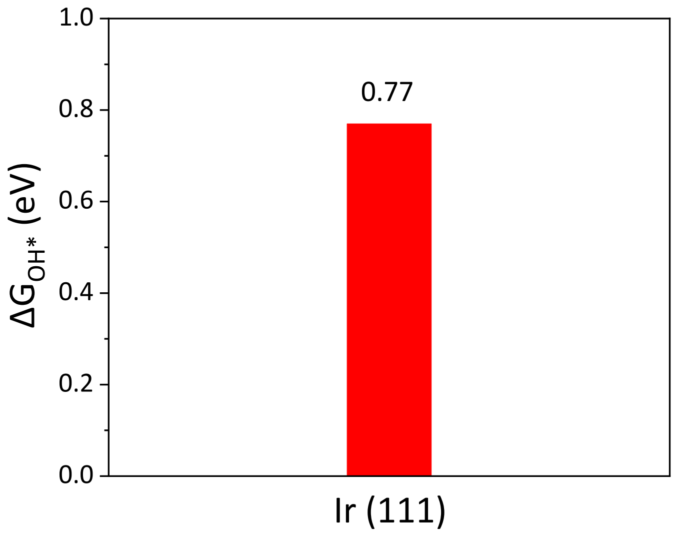

Supplementary Fig. 22 | OH adsorption energy. The difference on Gibbs free energy of the adsorbed OH at Ir (111) facet.

Supplementary Note 2.

1) *d* band theory correlation. As can be seen in supplementary Fig. 23-24, the *d* band center of the Ir at pure IrMo (001) locates closer to the Fermi level than that of the Ir (111), leading to a stronger H adsorption. The modification of OH spectator at Mo site of the IrMo (001) would shift the Ir *d* band center to a more negative position than those of the pure IrMo (001) and Ir (111). As shown in supplementary Fig. 23b, a linear correlation is observed between the calculated ΔG_{H^*} and *d* band center, which can be explained by the *d*-band theory that the negative shift of the d band center would weaken the H adsorption ability[2].

2) Sabatier Volcanos correlation. Also, the measured specific HER activity of the IrMo/CNT, Ir/C and Pt/C in acid condition are relationalized with their calculated ΔG_{H^*}. As shown in supplementary Fig. 23a, a good linear correlation is observed between the HER activity and the calculated ΔG_{H^*}. According to the Sabatier Principle, neither too strong nor too weak of the H adsorption is beneficial for facilitating the HER. The trend of the plot in supplementary Fig. 23a matched with the strong-adsorption side of the traditional HER Sabatier volcano plot[3].

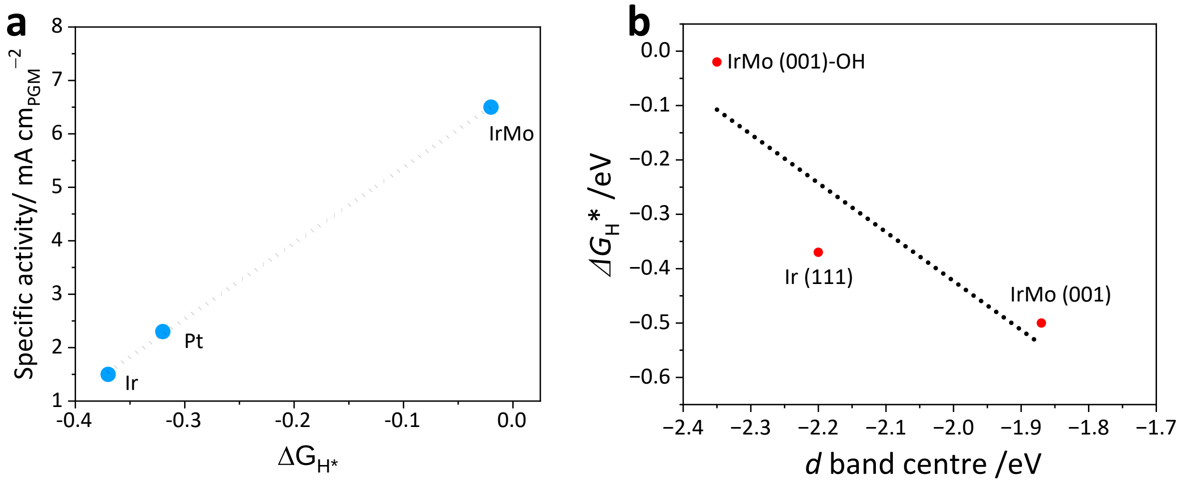

Supplementary Fig. 23 | Sabatier and *d* band theory correlation. a, Plot of hydrogen evolution reaction activity (at η = 20 mV) in acid condition versus the ΔG_{H^*}. **b**, Plot of ΔG_{H^*} versus d band centre for pure IrMo (001), IrMo (001)-OH and Ir (111) slab.

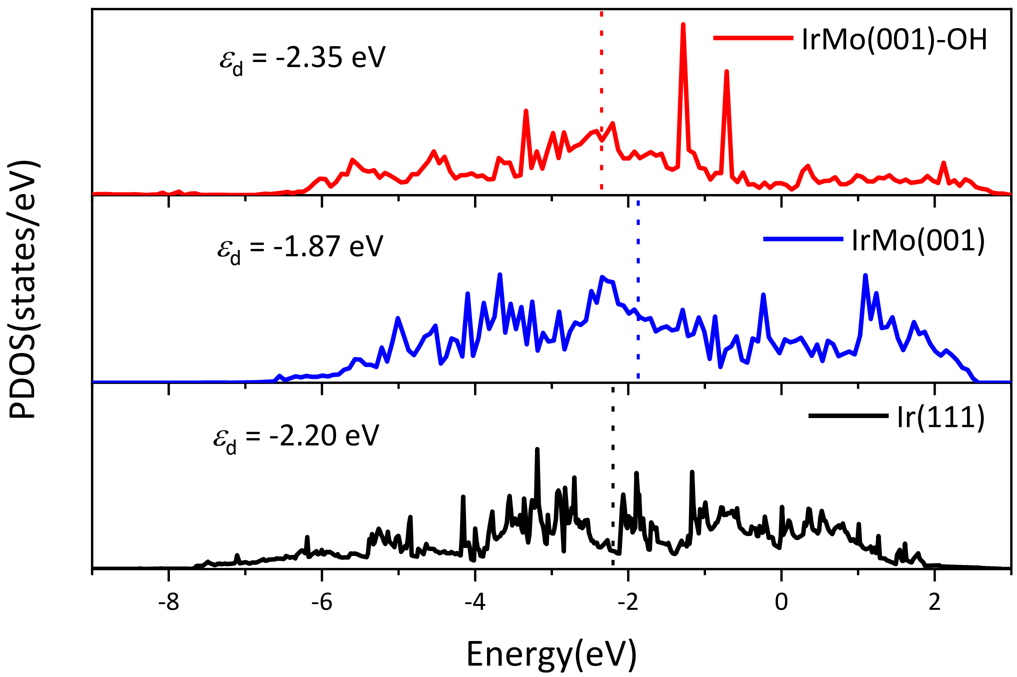

Supplementary Fig. 24 | Density of state analysis. The partial density of states (PDOS) of the Ir *d* state at pure IrMo (001), IrMo (001)-OH and Ir (111).

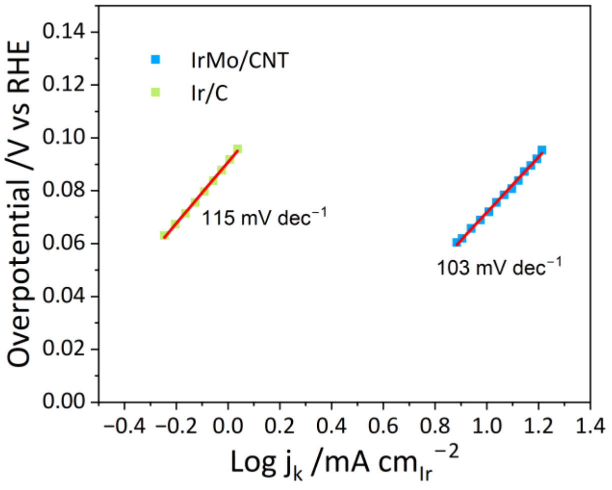

Supplementary Fig. 25 | Tafel approximation. Intrinsic Tafel plots of IrMo/CNT and Ir/C in the region of the overpotential greater than 60 mV. The Tafel slopes are used for Tafel approximation to identify the HER reaction pathway.

Supplementary Table 1. Comparison of the reported noble metal-based electrocatalysts on alkaline HER activity

Catalyst	Electrolyte	TOF at η=50 mV / H_2 s^{-1}	Tafel slope / mV dec^{-1}	Overpotential at 10 mA cm_{geo}^{-2} / mV	Reference
IrMo intermetallic	1 M KOH	15.4	22	17	This work
$IrMo_{0.59}$ alloy	1 M KOH	–	50	23	[4]
IrCo@NC-500	1M KOH	–	80	45	[5]
DSIrNi	1 M KOH	–	48	17	[6]
Li-$IrSe_2$@CNT	1 M KOH	0.03	–	72	[7]
Ir@CON	1 M KOH	~0.66	29	14	[8]
Ru black	1 M NaOH	1.04	80	125	[9]
RuCo alloy	1 M KOH	~1.5	39	40	[10]
RuMo alloy	1 M KOH	3.6	25	18	[11]
Pt_2Ni_3-P	1 M KOH	–	66	44	[12]
Pt–Ni	0.1M KOH	–	59	~60	[13]

Supplementary Table 2. Surface energy of different exposed facet at IrMo intermetallic

Surface	Structure (side view)	Termination	Surface energy (J m^{-2})
100	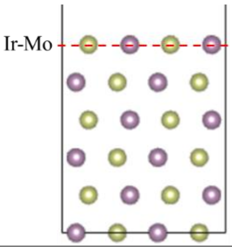	Ir-Mo	2.42
010	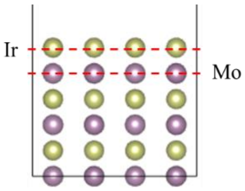	Ir	3.08
		Mo	2.96
001	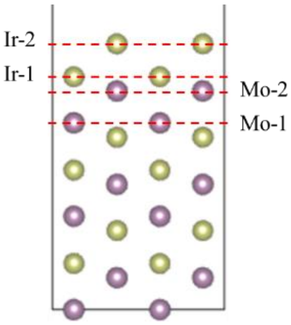	Ir-1	2.22
		Ir-2	3.52
		Mo-1	2.61
		Mo-2	3.52
011	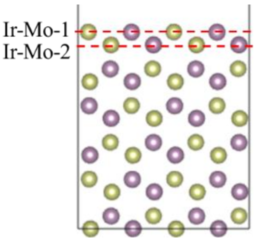	Ir-Mo-1	2.58
		Ir-Mo-2	3.05
101	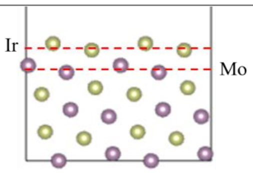	Ir	2.84
		Mo	2.87
111	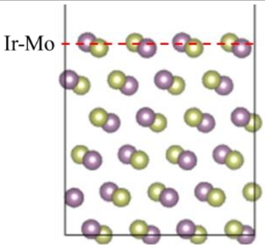	Ir-Mo	2.72

Supplementary Table 3. The calculated *ΔG* of the reaction intermediates for alkaline HER

intermediates\slab	IrMo (001)-OH	Ir (111)
H_2O*	0.019 eV	-0.026
H* + HO*	-0.051	0.215
H*	-0.017	-0.380

Supplementary Reference

1 Zhang, J. *et al.* Composition-Tunable Antiperovskite CuxIn1-xNNi3 as Superior Electrocatalysts for the Hydrogen Evolution Reaction. *Angew. Chem. Int. Ed.* **132**, 17641-17646 (2020).

2 Chen, H. *et al.* Promoting Subordinate, Efficient Ruthenium Sites with Interstitial Silicon for Pt-Like Electrocatalytic Activity. *Angew Chem Int Ed Engl* **58**, 11409-11413 (2019).

3 Nørskov., J. K. *et al.* Trends in the Exchange Current for Hydrogen Evolution. *J. Electrochem. Soc.* **152**, 23-26 (2005).

4 Fu, L. *et al.* IrMo Nanocatalysts for Efficient Alkaline Hydrogen Electrocatalysis. *ACS Catal.* **10**, 7322-7327 (2020).

5 Jiang, P. *et al.* Tuning the Activity of Carbon for Electrocatalytic Hydrogen Evolution via an Iridium-Cobalt Alloy Core Encapsulated in Nitrogen-Doped Carbon Cages. *Adv. Mater.* **30**, 1705324 (2018).

6 Liu, S. *et al.* Dislocation-Strained IrNi Alloy Nanoparticles Driven by Thermal Shock for the Hydrogen Evolution Reaction. *Adv. Mater.* **32**, 2006034 (2020).

7 Zheng, T. *et al.* Intercalated Iridium Diselenide Electrocatalysts for Efficient pH-Universal Water Splitting. *Angew. Chem. Int. Ed.* **58**, 14764-14769 (2019).

8 Mahmood, J. *et al.* Encapsulating Iridium Nanoparticles Inside a 3D Cage-Like Organic Network as an Efficient and Durable Catalyst for the Hydrogen Evolution Reaction. *Adv. Mater.* **30**, 1805606 (2018).

9 Creus, J. *et al.* Ligand-Capped Ru Nanoparticles as Efficient Electrocatalyst for the Hydrogen Evolution Reaction. *ACS Catal.* **8**, 11094-11102 (2018).

10 Mao, J. *et al.* Accelerating water dissociation kinetics by isolating cobalt atoms into ruthenium lattice. *Nat. Commun.* **9**, 4958 (2018).

11 Tu, K. *et al.* A Novel Heterostructure Based on RuMo Nanoalloys and N-doped Carbon as an Efficient Electrocatalyst for the Hydrogen Evolution Reaction. *Adv. Mater.* **32**, 2005433 (2020).

12 Wang, P., Shao, Q., Guo, J., Bu, L. & Huang, X. Promoting Alkaline Hydrogen Evolution Catalysis on P-Decorated, Ni-Segregated Pt–Ni–P Nanowires via a Synergetic Cascade Route. *Chem. Mater.* **32**, 3144-3149 (2020).

13 Kavian, R. *et al.* Pt–Ni octahedral nanocrystals as a class of highly active electrocatalysts toward the hydrogen evolution reaction in an alkaline electrolyte. *J. Mater. Chem. A* **4**, 12392-12397 (2016).
